# Evolutionary Change within a Bipotential Switch Shaped the Sperm/Oocyte Decision in Hermaphroditic Nematodes

**DOI:** 10.1371/journal.pgen.1003850

**Published:** 2013-10-03

**Authors:** Yiqing Guo, Xiangmei Chen, Ronald E. Ellis

**Affiliations:** Department of Molecular Biology, Rowan-SOM and the UMDNJ-SOM, B303 Science Center, Stratford, New Jersey, United States of America; University of California San Diego, United States of America

## Abstract

A subset of transcription factors like Gli2 and Oct1 are bipotential — they can activate or repress the same target, in response to changing signals from upstream genes. Some previous studies implied that the sex-determination protein TRA-1 might also be bipotential; here we confirm this hypothesis by identifying a co-factor, and use it to explore how the structure of a bipotential switch changes during evolution. First, null mutants reveal that *C. briggsae* TRR-1 is required for spermatogenesis, RNA interference implies that it works as part of the Tip60 Histone Acetyl Transferase complex, and RT-PCR data show that it promotes the expression of *Cbr-fog-3*, a gene needed for spermatogenesis. Second, epistasis tests reveal that TRR-1 works through TRA-1, both to activate *Cbr-fog-3* and to control the sperm/oocyte decision. Since previous studies showed that TRA-1 can repress *fog-3* as well, these observations demonstrate that it is bipotential. Third, TRR-1 also regulates the development of the male tail. Since *Cbr-tra-2 Cbr-trr-1* double mutants resemble *Cbr-tra-1* null mutants, these two regulatory branches control all *tra-1* activity. Fourth, striking differences in the relationship between these two branches of the switch have arisen during recent evolution. *C. briggsae trr-1* null mutants prevent hermaphrodite spermatogenesis, but not *Cbr-fem* null mutants, which disrupt the other half of the switch. On the other hand, *C. elegans fem* null mutants prevent spermatogenesis, but not *Cel-trr-1* mutants. However, synthetic interactions confirm that both halves of the switch exist in each species. Thus, the relationship between the two halves of a bipotential switch can shift rapidly during evolution, so that the same phenotype is produce by alternative, complementary mechanisms.

## Author Summary

In roundworms, the transcription factor TRA-1 controls sexual development. We show that TRR-1 is part of a complex of proteins that adds acetyl groups to its targets, and that this complex works with TRA-1 to initiate spermatogenesis. By contrast, a truncated form of TRA-1 blocks spermatogenesis. Because two different forms of this transcription factor oppose each other, TRA-1 is bipotential. To see if the interdependent relationship between these two forms has affected evolutionary change, we compared related species of roundworms. In one species, mutations that affect TRR-1 cause all germ cells to become oocytes, but mutations that affect three FEM proteins, which also regulate TRA-1, do not. In the other species, the roles of these regulatory genes are reversed. Thus, the relative importance of the two halves of this bipotential switch has changed during recent evolution.

## Introduction

Most animals carry the genetic information needed to produce two alternate sexes, and must choose which program to employ. In the nematode *C. elegans*, for example, a signal transduction pathway controls the activity of the master transcription factor TRA-1 to determine sex [reviewed by 1], [2], [3]. The leading model is that hermaphrodites are produced when TRA-1 prevents the expression of male genes like *fog-3* in germ cells [4], *egl-1* in the HSN neurons [5], and *mab-3* in the intestine and tail [6]. TRA-1 activity is itself controlled by interactions between the TRA-2 receptor and a complex of FEM proteins, which target TRA-1 for degradation [7]. As a result, *XX* animals have abundant TRA-1, which causes hermaphrodite development, and *XO* animals lack TRA-1, which allows male development.

In this model, the switch that controls sex-determination is part of a linear pathway. However, the fact that TRA-1 is a Gli protein [8] implies that its regulation might be more complex. Mammalian Gli proteins and their *Drosophila* homolog Ci are not only cleaved to form repressors or ubiquitinylated and degraded [9], but also function as activators [10]–[12]. As a consequence, these proteins constitute a key class of bipotential transcription factors [13].

Some of these regulatory interactions are also found in nematodes. For example, TRA-1 can also be cleaved to form a repressor [14], or eliminated by ubiquitinylation and degradation [7]. Although it is not known if TRA-1 is a direct activator of any male genes, older *tra-1* mutants cannot maintain spermatogenesis [15], [16]. This phenotype and several other traits make the germ line an ideal tissue for elucidating how TRA-1 functions. First, both *C. elegans* and the related nematode *C. briggsae* make *XX* hermaphrodites, which resemble females but produce sperm before beginning oogenesis. Thus, both sexual fates can be studied in a single animal. Second, self-fertilization simplifies genetic screens, a fact that has been used for decades in *C. elegans*, and is now being exploited in *C. briggsae* [17]. Third, information about germ cell development in *C. elegans* provides the background needed for dissecting TRA-1 regulation [reviewed by 2]. Fourth, although some TRA-1 mutations promote spermatogenesis and others promote oogenesis, TRA-1 is not absolutely required for either fate, since null mutants make both sperm and oocytes [15], [16]. Thus, we can isolate mutations that affect all aspects of *tra-1* activity by observing how they alter the sperm/oocyte decision.

This system is also a leading model for studying evolution. Hermaphroditic reproduction originated on several independent occasions in the genus *Caenorhabditis* [18]–[20]. It appears to have required regulatory changes that affect both sex-determination and sperm activation [21]. Hence, comparative analyses of *C. briggsae* and *C. elegans* could elucidate not only how TRA-1 functions, but also how the sex-determination pathway changed during recent evolution.

Here, we show that TRR-1, the homolog of mammalian TRRAP (TRansformation/tRanscription domain-Associated Protein), acts as part of the Tip60 Histone Acetyl Transferase (HAT) complex to control germ cell fates in *C. briggsae*. Genetic and molecular experiments imply that it works with TRA-1 as a co-activator to promote the expression of genes like *fog-3*. This activity occurs in parallel to the regulation of TRA-1 by the receptor TRA-2 and the FEM proteins, which control the levels of a cleaved TRA-1 repressor. Although these two functions have been conserved throughout *Caenorhabditis*, their relative importance has changed dramatically during recent evolution. Moreover, this shift shows that a novel trait can evolve by distinct but compensatory changes within the structure of a binary switch, and explains the puzzling differences between the functions of the *C. elegans* and *C. briggsae fem* genes [22].

## Results

### Identification of a new *fog* gene in *C. briggsae*

**Figure 1:**
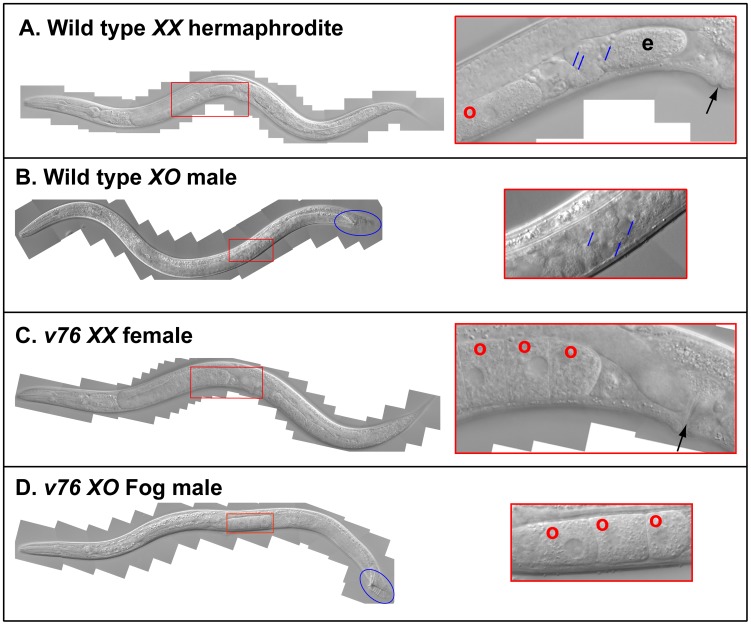
A new *C. briggsae* Fog gene. Young adult animals of the following genotypes were photographed using DIC optics. A. Wild-type *XX* hermaphrodite. B. Wild-type *XO* male. C. *v76 XX* female. D. *v76 XO* Fog male. Expanded images of the boxed regions are shown on the right. Anterior is left and ventral down. The hermaphrodite vulva is indicated with a black arrow, and the male tail with a blue oval. Oocytes are marked with a red “o”, sperm with a blue arrow, and a one-celled embryo in the wild type with and “e”.

**Figure 2:**
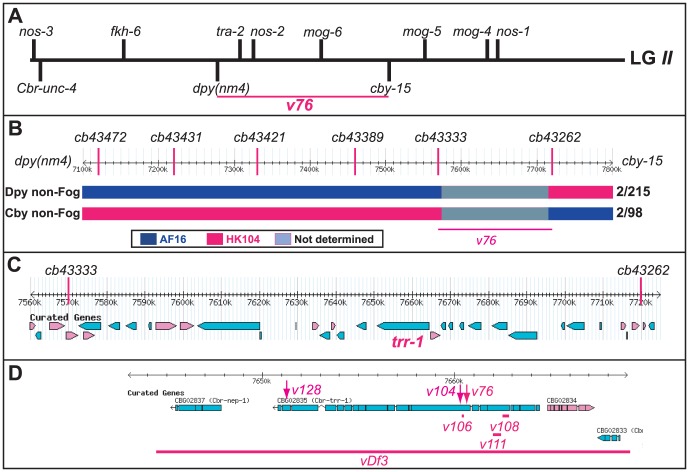
Cloning the *C. briggsae* Fog gene *trr-1* by SNP mapping. (A) Genetic map of LG*II*, showing the location of *trr-1* based on three-factor crosses (Methods). The positions of potential sex-determination genes on the top are from WormBase, and those of the marker genes are from www.briggsae.org. (B) The region extending from 7100 to 7800 kb and the position of each SNP were downloaded from WormBase. The structure of critical recombinant chromosomes is indicated by color, and the frequency of these recombinants is shown at the right. (C) The predicted genes in the region extending from 7560 to 7720 kb were downloaded from WormBase. (D) The predicted structure of *trr-1* was downloaded from WormBase, The location of each missense allele is marked by an arrow, and the extent of each deletion by a line.

In *C. elegans*, three *fog* genes were identified by screening for mutations that cause feminization of the germ line (the Fog phenotype) [23]–[25]. Because only two of these genes have homologs in *C. briggsae*, the regulation of germ cell fates must have changed during recent evolution [26]. When we screened for *C. briggsae* Fog mutants, we identified the new gene *Cbr-she-1* [17], and also recovered the mutations *v76* and *v104*. These alleles are recessive, fail to complement each other, and affect germ cell fates in both sexes — the *XX* animals only make oocytes and develop as females, and the *XO* males make oocytes instead of sperm, but are otherwise normal (Fig. 1). Thus, this gene controls the sperm/oocyte decision. Surprisingly, it maps to the left arm of chromosome *II* (Methods, Fig. 2A), which contains no homologs of *C. elegans fog* genes.

### TRR-1 regulates germ cell fates in *C. briggsae*

We used SNP mapping to clone this gene. Using the linked mutations *Cbr-cby-15(sy5148)* and *Cbr-dpy(nm4)*, we identified recombinants between the AF16 and HK104 strains, and located each breakpoint using SNPs (Fig. 2B). Next, we studied a 150 kb region of the *C. briggsae* genome [27] that contained the new gene (Fig. 2C), and used RNA interference to test candidates. Knocking down *Cbr-trr-1* activity caused a Fog phenotype. Finally, we sequenced *Cbr-trr-1* genomic DNA, and found that both *v76* and *v104* were missense mutations (Fig. 2D, Table S1). Hence, *C. briggsae trr-1* is a *fog* gene that regulates the decision of germ cells to become sperm or oocytes. It encodes the nematode homolog of the human TRRAP protein, which is a component of several Histone Acetyl Transferase (HAT) complexes [28].

Finally, we used RT-PCR and RACE to clone a complete *Cbr-trr-1* transcript, which differs slightly from that predicted at WormBase (Fig. S1). The encoded protein is 4037 amino acids long, and is 67% identical and 82% similar to *C. elegans* TRR-1.

### TRR-1 is essential for spermatogenesis and for embryonic development

Because these alleles of *Cbr-trr-1* were missense mutations, it was possible that neither caused a complete loss of function. Thus, we used a non-complementation screen to identify additional alleles (Fig. S2, Table S1). All of the new mutations were also Fog when homozygous, with the exception of the large deletion *vDf4*, which caused embryonic lethality. The Fog alleles include three small deletions, two of which shift the reading frame, and the deletion *vDf3*, which removes the entire gene (Fig. 2D). We conclude that eliminating *Cbr-trr-1* causes the production of oocytes instead of sperm. Although all *Cbr-trr-1* males made oocytes, 34% of *v108* males and 44% of *vDf3* males produced a few sperm before beginning oogenesis.

TRR-1, like human TRRAP [28], contains a PI-kinase like domain with an inactive catalytic site near its carboxyl terminus. This domain is altered by the new *v128* missense mutation, which demonstrates its importance for TRR-1 activity.

Although *C. elegans trr-1* regulates vulval development [29], none of the *C. briggsae* mutants showed vulval defects. Furthermore, *Cbr-trr-1(RNAi) Cbr-lin-8(RNAi)* animals had normal vulvae, which suggests that *C. briggsae trr-1* is not a synthetic multivulva gene.

Finally, we looked for a maternal requirement by crossing homozygous females with heterozygous males. About 33% of the *Cbr-trr-1(v76)* progeny died as embryos, as well as all of the *Cbr-trr-1(v108)* progeny and some of the *v108* heterozygotes (Table S2A). Since *v108* is a deletion, this represents the null phenotype. To test for a strict maternal effect, we crossed homozygous females with wildtype males, and saw that some of their embryos died and some survived (Table S2B). Taken together, these results show that maternal and zygotic TRR-1 work together to promote viability during embryogenesis. In adults, the sperm/oocyte decision is primarily controlled by zygotic TRR-1.

### TRR-1 acts through the Tip60 HAT complex to regulate germ cell fates

TRR-1 is the sole nematode TRRAP protein [29]. In eukaryotes, TRRAP proteins are components of both the GNAT and MYST families of HAT complexes [reviewed by 28]. To see if TRR-1 acts through either of these complexes to control germ cell fates, we used RNA interference to knock down other components in *C. briggsae* (Table 1). Targeting *Cbr-pcaf-1*, which encodes the catalytic component of the GNAT family, did not affect germ cell fates, and knocking down three other components of this HAT complex also failed to produce Fog animals. However, targeting other components of the Tip60 HAT complex caused phenotypes like that of *Cbr-trr-1*. Knocking down four genes, including the catalytic component *Cbr-mys-1*, produced Fog animals at high frequency, and four other genes produced Fog animals at low frequency. For some genes, we saw embryonic lethality and some sterility. Thus, TRR-1 acts through the Tip60 HAT complex to control the sperm/oocyte decision in *C. briggsae*.

**Table 1:** Components of the Tip60 HAT complex control the sperm/oocyte decision.

A	PCAF/SAGA HAT Complex (GNAT family)		
Human homolog	Yeast homolog	*C. briggsae* gene	RNAi Phenotype
**PCAF**	**GCN5**	***pcaf-1 I***	**WT**
ADA2a	Ada2	*ada-2 II*	WT
TAF6L	TAF6	*taf-6.1 II*	Emb, Ste
TAF9	TAF9	*taf-9 III*	Emb

B	Tip60/NuA4 HAT Complex (MYST family)		
Human homolog	Yeast homolog	*C. briggsae* gene	RNAi Phenotype
**Tip60**	**Esa1**	***mys-1 V***	**Fog**
Epc1	Epl1	*epc-1 III*	Fog, Emb
ING3	Yng2	*ing-3 II*	WT
P400	Eaf1	*ssl-1 III*	Fog (rare), Emb
DMAP	Eaf2	*ekl-4 I*	Fog (rare), Emb
GaS51	Yaf9	*gfl-1 IV*	Fog
RuvBL1	Rvb1	*ruvb-1 V*	Fog (rare), Emb, Ste
RuvBL2	Rvb2	*ruvb-2 IV*	Fog (rare), Emb, Ste
Mrg15	Eaf3	*mrg-1 III*	WT
YL-1	Vps72	*CBG03742 I*	Fog, Ste

A. Analysis of the PCAF/SAGA HAT complex. B. Analysis of the Tip60/NuA4 HAT complex. The phenotypes are Fog (feminization of the germ line), Emb (embryonic lethal) and Ste (Sterile). *C. briggsae* genes were identified by BLAST. For details, see methods and Table S5.

### The Tip60 complex acts through TRA-1 to promote spermatogenesis

To determine where the Tip60 complex acts in the sex-determination pathway, we examined double mutants. First, we studied the upstream genes *tra-2* and *tra-3*, which promote female development. Mutations in either gene cause *XX* animals to produce only sperm, and transform the body towards male fates [30]. Moreover, *Cbr-tra-2(nm1)* is a nonsense mutation that should act as a null allele, so it is ideal for epistasis. In each case, double mutants with *Cbr-trr-1* restored the ability of *XX* animals to produce oocytes (Table S3), which indicates that *Cbr-trr-1* acts downstream of these genes, or in parallel to them.

Next we studied mutations in the *Cbr-fem* genes, which are required for male somatic development. Although these genes act downstream of *tra-2*, null mutants produce sperm and oocytes, and develop as normal hermaphrodites [22]. As expected, double mutants with *Cbr-trr-1* caused *XX* animals to produce only oocytes (Table S3). In addition, we saw a synthetic lethal interaction between *Cbr-trr-1* and *Cbr-fem-2*. FEM-2 is a protein phosphatase [31], [32], which also causes synthetic lethality in *C. elegans*, in combination with *Cel-mel-11* mutations [33].

**Figure 3:**
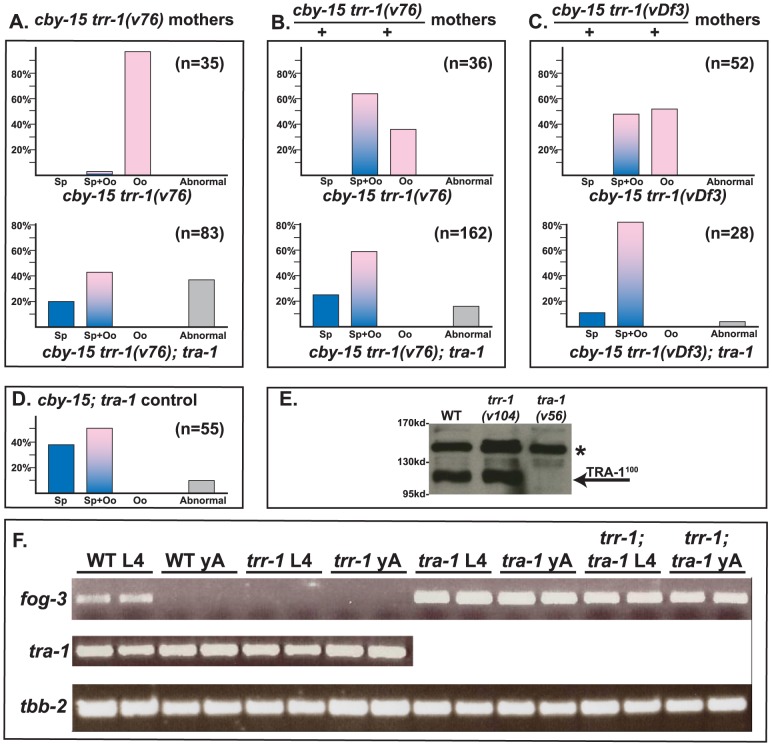
TRR-1 acts through TRA-1 to promote spermatogenesis and *fog-3* expression. A–D. The null allele *tra-1(nm2)* is epistatic to mutations in *trr-1*. Animals in A were produced by *trr-1(v76)* mothers, except for the *cby-15; tra-1* control at the bottom. Those in B and C were produced by self-fertilization from heterozygous mothers. Blue colored bars indicate spermatogenesis, pink bars indicate oogenesis, and mixed colors represent animals that make both sperm and oocytes. Abnormal germ lines are gray. E. Western blot showing that the levels of TRA-1^100^ are not altered in a *trr-1* mutant. The absence of TRA-1^100^ in a *tra-1(v56)* mutant served as a negative control. (Full-length TRA-1 at the top is obscured by a non-specific band, marked by a star). F. RT-PCR analyses of hand-picked worms. Each age and genotype was run with independent samples, which are presented side-by-side.

Finally, we studied the interaction of *Cbr-trr-1* with *Cbr-tra-1*, the master regulator of sexual development in nematodes (Fig. 3A–D). We used the *Cbr-trr-1* deletion *vDf3* and the point mutation *v76*, which has a strong germline phenotype but causes only low levels of embryonic lethality, so that we could study homozygous children of homozygous mothers. In all cases, we found that the *Cbr-tra-1(nm2)* mutation restores the production of sperm to *Cbr-trr-1* mutants. This *tra-1* allele is a nonsense mutation and behaves like a null allele [30]. Thus, the Tip60 complex promotes spermatogenesis, but only if TRA-1 is functional.

### TRR-1 works through TRA-1 to promote the expression of *fog-3*

In *C. elegans*, TRA-1 acts directly on the *fog-3* promoter [4], and these binding sites have been conserved in *C. briggsae* [34]. Moreover, *C. briggsae fog-3* is required for germ cells to become sperm, and the level of *fog-3* transcripts is correlated with spermatogenesis [34]. Since TRRAP proteins regulate transcription, we studied *Cbr-tra-1* and *Cbr-fog-3* transcripts, to see if either was affected by inactivation of *Cbr-trr-1*. We could detect no change in *Cbr-tra-1* transcript levels or protein levels in *Cbr-trr-1* mutants (Fig. 3E, F), so the Tip60 complex does not control the expression of TRA-1. However, loss of *Cbr-trr-1* activity eliminates *fog-3* transcripts, but only if TRA-1 is active (Fig. 3F). Thus, we propose that the Tip60 HAT complex cooperates with TRA-1 to increase *fog-3* expression and promote spermatogenesis.

### TRR-1 also works through TRA-1 to control development of the male tail

**Figure 4:**
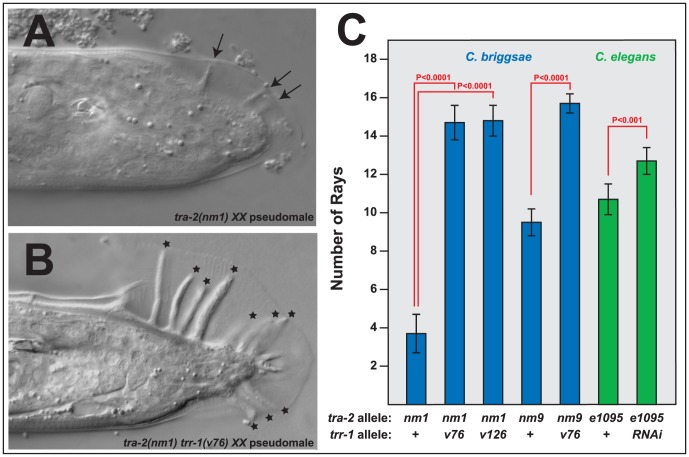
Mutations in *trr-1* and *tra-2* act synthetically to produce male tails. A, B. Photomicrographs of typical *XX* male tails, prepared using DIC optics. The arrows indicate the small, spindly rays typical of *tra-2* mutants, and the stars mark the large rays seen in *tra-2 trr-1* double mutants. C. Number of rays detected in single and double mutants. (The wild-type male produces 18 rays). Error bars represent 95% confidence intervals, and the indicated probabilities were calculated using the Mann Whitney U test.

Although the transmembrane receptor TRA-2 acts through TRA-1 to promote female cell fates, two differences between these genes have been conserved among *Caenorhabditis* species. First, *tra-2(null) XX* mutants do not make complete male tails, but instead produce stubby tails that lack many of the sensory structures known as rays (Figure 4A); by contrast, *tra-1(null)* mutants develop perfect male tails [30], [35]. Second, *tra-2(null)* mutants only produce sperm, whereas *tra-1(null)* mutants make sperm early in life and then switch to oogenesis [15], [16], [30]. These differences do not reflect the degree of transformation, since *tra-2(null)* mutants have more strongly masculinized germ lines, but less masculinized tails.

We were surprised to find that *Cbr-tra-2 Cbr-trr-1* double mutants resemble *Cbr-tra-1* mutants in both of these traits. The double mutants make oocytes (Table S3), which shows that the Tip60 complex acts downstream of *tra-2*, or in parallel to it. However, oogenesis usually begins after an initial burst of spermatogenesis, as in *Cbr-tra-1 XX* animals. Moreover, the double mutants develop much better male tails than those seen in *Cbr-tra-2* single mutants, with rays that are almost wildtype in both length (Figure 4B) and number (Figure 4C). Thus, the Tip60 complex is required for precisely the two *tra-1* phenotypes that are not dependent on *tra-2.* As a consequence, *Cbr-tra-2 Cbr-trr-1* double mutants appear to have lost all *tra-1* activity.

### The control of spermatogenesis by TRR-1 has been conserved in nematodes

The *trr-1* gene regulates vulval development in *C. elegans*, and the embryos produced by null mutants die [29]. To see if *Cel-trr-1* regulates the sperm/oocyte decision, we used RNA interference to knock it down in males, since the production of oocytes by a male would prove that a change in germ cell fates had occurred. At 20°C, 14% of *Cel-trr-1(RNAi)* males made oocytes (n = 58). This phenotype was sensitive to temperature, since 23% of males made oocytes at 25°C (n = 62), and an additional 8% failed to produce mature germ cells. Finally, knocking down *trr-1* activity in the male/female species *C. remanei* also caused males to produce oocytes (Fig. S3), so we believe that the male function of the Tip60 complex in germ cell fates has been conserved during *Caenorhabditis* evolution.

We also tested the ability of *C. elegans trr-1* to act synthetically with *tra-2*, by using RNA interference to knock down *Cel-trr-1* activity in *Cel-tra-2(e1095)* null mutants. As in *C. briggsae*, lowering *trr-1* activity produced better, more masculine tails in *tra-2 XX* animals (Fig. 4C). In addition, these double mutants also produced sperm and oocytes.

Finally, we tested *C. elegans* hermaphrodites. Rare *Cel-trr-1(RNAi) XX* animals developed as females at 25°C (6%, n = 135), although none of them did at 20°C (n = 332). This result suggested that *trr-1* influences the sperm/oocyte decision in *C. elegans* hermaphrodites, but that it plays a smaller role than in *C. briggsae*. To measure its relative importance in each species, we assayed gene activity in sensitive genetic backgrounds. First, we studied partial loss-of-function mutations in either *C. elegans fem-1* or *fem-2*. At permissive temperatures, these animals usually developed as hermaphrodites; however, if *Cel-trr-1* activity was knocked down by RNAi, they became female (Table 2). Thus, *trr-1* normally plays a minor role in *C. elegans* sex-determination because the activity of the *fem* genes is high.

**Table 2:** Tip60 and the FEM complex play complementary roles in germ line regulation.

A. *Cel-fem-1(hc17*ts*)*				
	Female	Hermaphrodite	Sterile	N
No RNAi	32%	68%	0%	50
*trr-1(RNAi)*	78%	9%	17%	517
*mys-1(RNAi)*	83%	17%	0%	127

B. *Cel-fem-2(b245*ts*)*				
	Female	Hermaphrodite	Lethal	N
No RNAi	6%	94%	0%	50
*trr-1(RNAi)*	36%	11%	48%	366

C. *Cbr-fem-3(nm63)*				
	Female	Hermaphrodite		N
*fem-3(nm63)*	0%	100%		*
*trr-1(v104); fem-3(nm63)* †	100%	0%		101
*trr-1(v104)* †	0%	100%		56
*trr-1(v128); fem-3(nm63)* ^††^	100%	0%		53
*trr-1(v128)* ‡	0%	100%		20

A. Knocking down Tip60 genes in a *Cel-fem-1(ts)* mutant. B. Knocking down Tip60 genes in a *Cel-fem-2(ts)* mutant. C. Analysis of double mutants using weak alleles of *trr-1* in *C. briggsae*.

* — [22];

† — The Cby progeny of *cby-15 trr-1(v104)/++* mothers;

‡ — The Cby progeny of *cby-15 trr-1(v128)/++* mothers.

Second, we studied partial loss-of-function mutations in *C. briggsae trr-1*. These mutants usually develop as hermaphrodites when maternal *trr-1* activity is present (Table 2). Furthermore, null alleles of the *C. briggsae fem* genes are famous for not preventing hermaphrodite development, and *Cbr-fem-3(nm63)* does not alter the number of sperm produced by hermaphrodites [22]. However, all germ cells in *Cbr-trr-1(weak); Cbr-fem-3(nm63)* double mutants developed as oocytes rather than sperm (Table 2), and experiments with *Cbr-fem-2* gave similar results. Thus, a loss of *fem* activity has no effect on *C. briggsae* hermaphrodites because *trr-1* activity is normally high. We conclude that the relative importance of each regulator has shifted during the independent evolution of these self-fertile hermaphrodites.

## Discussion

### Screens in related species can identify conserved regulatory proteins

A major thrust of evolutionary developmental biology has been comparing traits between model organisms like *C. elegans* or *Drosophila melanogaster* and closely related species [e.g. 17], [22], [36], [37]. These studies have focused on identifying key differences that provide a window into evolutionary mechanisms.

However, our results emphasize an equally important use of comparative evolutionary studies — conserved aspects of gene regulation are sometimes more easily detected in one species than in another. For example, we found that Fog mutations in the *trr-1* gene are common and easy to work with in *C. briggsae*. By contrast, the role that *trr-1* plays in spermatogenesis had not been detected in *C. elegans*, despite decades of work on sex-determination [reviewed by 3] and the existence of *C. elegans trr-1* deletion mutants [29].

What factors might account for this difference? First, mutations in essential genes might be detected more easily in one species than another, because their pleiotropic activities differ. Second, the absence of redundant regulatory pathways might make it easier to identify mutants in one species. Third, the relative importance of the genes might differ in each species. All of these factors may have delayed the identification of the role Tip60 plays in sex determination.

We also note that the combination of forward genetic screens in *C. briggsae* with cloning by SNP mapping provides a powerful, unbiased approach to studying evolutionary differences. This technique has already been used to clone one other novel *C. briggsae* gene [17]. In addition, SNP mapping has helped assign new mutations to known genes from several well-characterized pathways [38], [39].

### The Tip60 HAT complex works with TRA-1 to regulate cell fates in nematodes

In nematodes, *tra-1* plays a critical role in the regulation of sexual development. Null mutations in either *C. elegans* or *C. briggsae* cause *XX* animals to develop as fertile males [30], [35]; a transformation that involves many cell fates. Because TRA-1 also determines the sex of the distantly related nematode *Pristionchus pacificus*, this role is ancient [40]. Finally, TRA-1 is the sole nematode homolog of the Gli proteins from humans and of *Cubitus interruptus* from flies [8], and like them, it acts by regulating the transcription of numerous target genes [4]–[6].

The transmembrane receptor TRA-2 acts through TRA-1 to promote female development, but their effects differ in the germ line and male tail [15], [16], [35]. Thus, we were surprised to find that *tra-2 trr-1* animals do not look like *tra-2* mutants, but instead resemble *tra-1* null mutants — they produce excellent male tails and make oocytes after an initial burst of sperm production. Since mutations in *trr-1* and *tra-2* affect both the germ line and cells of the developing tail, each of these genes must play a general role in the regulation of TRA-1, rather than a tissue-specific function in implementing one particular cell fate.

### The Tip60 complex works with TRA-1 to activate target genes

Although Tip60 works through TRA-1, knocking down Tip60 activity causes oogenesis, a phenotype normally associated with *increased* TRA-1 activity [16]. Since numerous papers have already shown that TRA-1 can repress some male genes [4]–[6], we infer that it is bipotential and can also activate genes needed for spermatogenesis.

To begin, two genetic experiments imply that repression by TRA-1 is mediated by a cleaved form of the protein, known as TRA-1^100^ [14]. First, the *tra-1(e2272*stop) mutant encodes a truncated form of the protein slightly shorter than TRA-1^100^ [41]; when its transcripts are protected from nonsense-mediated decay, they direct female development and oogenesis [42]. Second, a truncated from of TRA-1 created by the duplication *eDp24* also directs female development [43]. Since the known targets of TRA-1 are male genes, truncated TRA-1 must work as a repressor. The levels of this repressor are protected by TRA-2, but decreased by three FEM proteins and CUL-2, which form part of a ubiquitin-ligase complex [7].

**Figure 5:**
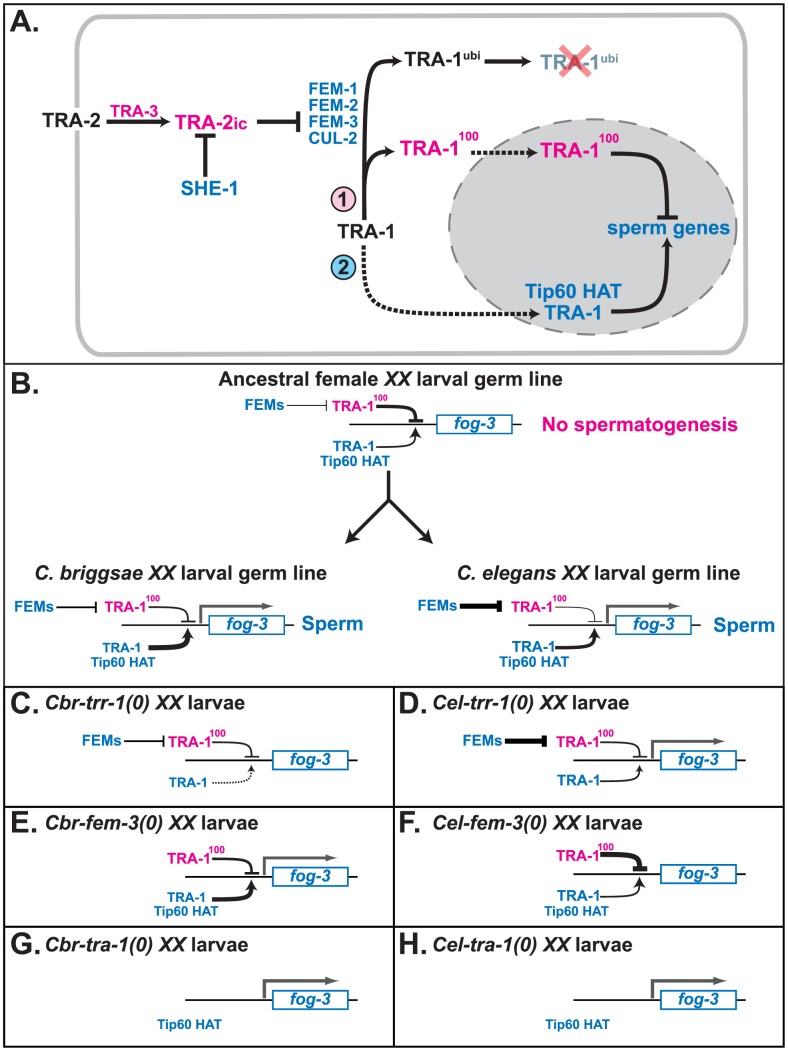
Model for how TRR-1 regulates sexual development. A. Proposed regulatory cascade for the specification of sexual fates in germ cells. Proteins promoting oogenesis are magenta, and those promoting spermatogenesis are blue. Arrows denote positive interactions or transformations, and lines with bars indicate negative interactions. Dotted lines mark import into the nucleus. B. Model for the convergent origin of self-fertility. In the ancestral species, the *fog-3* gene is inactive in *XX* larvae because TRA-1 repressing activity outweighs its activating activity. In *C. elegans, fog-3* is expressed because increased FEM activity lowers the amount of TRA-1 repressor in the larval germ line. By contrast, in *C. briggsae, fog-3* is expressed because of an increase in TRA-1 activating function. C–H. Models for how mutations in *C. briggsae* and *C. elegans* affect the expression of *fog-3* in larval hermaphrodites. The key factors are that the expression of *fog-3* is determined by a competition between TRA-1 activating and repressing activities, that in the complete absence of TRA-1, *fog-3* will be transcribed, and that in *C. elegans* either maternal TRR-1 activity, or an unknown chromatin regulator, allows for spermatogenesis in *trr-1* mutants. C. *Cbr-trr-1(0)*. D. *Cel-trr-1(0)*. E. *Cbr-fem-3(0)*. F. *Cel-fem-3(0)*. G. *Cbr-tra-1(0)*. H. *Cel-tra-1(0)*.

Previous studies hinted that TRA-1 has a second activity. First, wild-type males produce normal levels of full-length TRA-1, but lack the cleaved form [14]. Since old *tra-1* males begin producing oocytes, full-length TRA-1 appears necessary to maintain spermatogenesis [15], [16]. Second, eliminating the TRA-1 binding sites from the promoter of *C. elegans fog-3* prevents the transgene from directing spermatogenesis [4]. Taken together, these results imply that full-length TRA-1 might promote the expression of genes that activate spermatogenesis, just as full-length Gli proteins and Cubitus interruptus also work as activators [11], [12]. By identifying a co-factor required for the expression of *fog-3*, our current studies go farther. TRR-1 activity is needed for the expression of *fog-3*, but the loss of TRR-1 has no effect when TRA-1 is missing. The simplest explanation is that TRR-1 and the Tip60 HAT complex work with TRA-1 to promote the expression of *fog-3*, and that without Tip60 this activity is lost (Fig. 5). Because TRA-1 represses *fog-3* in other circumstances [4], this bipotential regulation resembles the control of *dpp* by cleaved and full-length Cubitus interuptus [11]. Furthermore, Ci and Gli3 use CREB-binding protein as a co-activator [44], [45], a protein that also has intrinsic HAT activity.

We suspect that the specificity of the *trr-1* phenotype in *C. briggsae* is due to two factors. First, the requirement for TRR-1 in embryogenesis is fully met by maternal supplies, so the homozygous mutants grow to adulthood. Second, TRA-1 only activates a few genes during development, so the *trr-1* sex-determination defect is very specific.

Finally, two types of models could explain how TRR-1 and the Tip60 complex regulate gene expression. On the one hand, they might acetylate histones in target promoters, to open up chromatin conformation and promote transcription [28]. Indeed, several studies indicate that histone acetylation could be a part of the sex-determination process in nematodes. For example, NASP-1, a histone chaperone, and HDA-1, a histone deacetylase, work with TRA-4 to control sexual fates in *C. elegans* [46]. Moreover, TRA-1 interacts with SynMuv B genes in the development of the *C. elegans* vulva [47]; and many of these genes regulate chromatin structure [reviewed by 48]. Finally, a polymorphism in *C. elegans* NATH-10, an acetyltransferase, affects several reproductive traits, including the number of sperm made by hermaphrodites [49]. Although a broad survey of histone modifications in *C. elegans* did not uncover significant levels of acetylation in histones of the *fog-3* promoter during larval development [50], only about 5% of the cells in these animals are becoming spermatocytes, so these experiments might not have been sensitive enough to detect variation in the developing germ line.

On the other hand, HAT complexes sometimes directly acetylate transcription factors, and the TRA-1 homologs Gli1 and Gli2 are acetylated in mammalian cells [51]. Thus, the Tip60 complex might directly acetylate TRA-1, to promote the activation of specific targets. Distinguishing these two models will require new methods for purifying TRA-1 complexes.

### The control of TRA-1 by the Tip60 complex has shifted during recent evolution

During evolution, some changes that accumulate in underlying regulatory pathways do not affect the phenotype [52]. In microevolution, this can produce populations with subtle differences in their regulatory architecture. For example, genetic variation that affects sex determination in *C. elegans* was revealed by different responses to the weak *tra-2* allele *ar221* [53], and genetic variation that affects vulval development was detected with a variety of weak mutations in the Ras pathway [54]. In macroevolution, this process can lead to species where significant regulatory differences underlie similar phenotypes. For example, the nematode vulva is induced by an EGF signal in *C. elegans*, but by a Wnt signal in *Pristionchus pacificus* [55], a distinction that involves many regulatory changes [56]. Because of these effects, mutations in orthologous genes sometimes have slightly different phenotypes in related species. For example, the Axin homolog PRY-1 plays similar roles in the development of the *C. elegans* and *C. briggsae* vulva, but the *pry-1* mutant phenotypes in these species are not identical [38].

In theory, the internal constraints found in bipotential switches could prevent the accumulation of such changes. Indeed, several aspects of the TRA-1 switch have remained stable during *Caenorhabditis* evolution. For example, null mutants of *tra-1* have similar phenotypes in both *C. elegans* and *C. briggsae* [15], [16], [30]. Moreover, the *fog-3* gene is a conserved target of TRA-1 that promotes spermatogenesis in each species [4], [34]. Finally, FEM-3 not only regulates TRA-1 activity, but can influence germ cell fates by acting on a separate, downstream target in each species [57], [58]. Under these constraints, what types of regulatory change are possible?

We addressed this issue by studying mutations in genes that control the activating and repressing activities of TRA-1, and found that the relative importance of the Tip60 HAT complex and the TRA-2/FEM degradation pathway has shifted during recent evolution. In *C. elegans*, null alleles in the *fem* genes transform *XX* animals into females [57], but a null allele of *trr-1* does not [29]. By contrast, null alleles have the opposite effects in *C. briggsae*. However, double mutants using weak alleles cause synthetic feminization in both species.

Although variation in sex-determination genes exists in nematode populations [59], there is no evidence it influences sexual development, and we observed no unexpected genetic interactions when mapping *trr-1* mutations using different wild isolates. Thus, we believe that these differences between *C. elegans* and *C. briggsae* do not involve *intra-*specific variation, but instead represent unique solutions to the problem of hermaphrodite development.

We propose that the ancestor of these nematodes used Tip60 to regulate TRA-1's activator function, and the FEM genes to control the levels of the TRA-1^100^ repressor (Fig. 5B). During the independent evolution of self-fertility in *C. elegans* and *C. briggsae*, changes within this switch helped promote *fog-3* expression and spermatogenesis in the *XX* animals of each species. We infer that *C. elegans* primarily relied on increased activity of the FEM proteins to eliminate TRA-1^100^ repressor in the larval germ line. This change required the novel FOG-2 protein, which increases FEM activity by down-regulating the translation of *tra-2* [24], [26]. By contrast, *C. briggsae* might have relied primarily on increasing the activating activity of TRA-1 and the Tip60 complex.

### The evolution of self-fertile hermaphrodites

Finally, the bipotential nature of TRA-1 might have influenced the origin of self-fertility in nematodes. Hermaphrodites evolved from male/female ancestors several times in the genus *Caenorhabditis* [20], and this transition can be modeled in the laboratory [21]. By contrast, large groups like the insects have not produced hermaphrodites. We suggest that the mutually antagonistic roles of TRA-1 in the sperm/oocyte decision might help explain this difference. In male/female species of worms, full-length and cleaved TRA-1 both have to be present in *XX* females. If small changes in the regulation of TRA-1 changed the balance between its activating and repressing activities, they might cause *XX* animals to make sperm, one of the changes needed to produce hermaphrodites. Furthermore, the complex web of regulators that control TRA-1 might have increased the opportunity for mutations that affect this switch, causing *XX* animals to make both sperm and oocytes. Thus, the structure of this regulatory switch might bias nematodes towards the evolution of self-fertility.

## Materials and Methods

### Strains

Strains were maintained as described by Brenner [60]. Wild type strains were AF16 and HK104. Mutant alleles were: *tra-1(nm2), tra-2(nm1), tra-2(nm9), tra-3(ed24ts)* [30], *fem-1(nm27), fem-3(nm63)* [22], *dpy(nm4)* [30], and *cby-15(sy5148)* (P. Sternberg, personal communication).

The *tra-1(v56)* allele was isolated while screening for *she-1* mutations. Homozygous *XX* mutants have male bodies but make oocytes. The lesion is a 7 bp deletion, removing nucleotides 1244 to 1250 of the *tra-1a* coding region. Because it causes a frame shift and early stop, it was used in western blots, and no *v56* protein was detected.

### Mapping

We counted self-progeny from *cby-15 trr-1(v76)/+ +* mothers, and observed 1044 wild type, 316 Cby Fog, 2 Fog, and 8 Cby hermaphrodites, implying a distance of 0.73 cM. We also counted self- progeny from *trr-1(v76) dpy(nm4)/+ +*, and observed 2303 WT, 698 Dpy Fog, 11 Fog, and 10 Dpy hermaphrodites, implying a distance of 0.69 cM.

### Mutant screens

Two mutants were isolated in screens for F_2_ females [17]. In the non-complementation screen (Fig. S1), we treated *cby-15 tra-2/dpy(nm4) trr-1(v76)* animals with 30 µg/ml trimethyl psoralen (TMΨ) followed by UV irradiation [61], or with 0.5% ethylmethane sulfonate [EMS, 60]. The P_0_ animals were transferred to new plates daily, and their F_1_ progeny screened for non-Dpy females. Each identified F_1_ female was crossed with wildtype males, and their F_2_ hermaphrodite progeny grown on individual plates. Those segregating F_3_ Cby Fog pseudo-males were identified and crossed with wildtype males. Finally, we screened the progeny of these crosses for recombinant Cby Fog animals that had lost the *tra-2* mutation, and backcrossed each at least ten times.

### SNP mapping

We mapped *v76* with SNPs that differ between the strains AF16 and HK104, as described [17]. Cby non-Fog recombinants were identified among the progeny of *trr-1 cby-15* [AF16]*/+ +* [HK104] mothers, and Dpy non-Fog recombinants from *dpy(nm4) trr-1* [AF16]*/+ +* [HK104] mothers. After establishing homozygous lines, we used the PCR to amplify and score DNA near each SNP from each of the recombinants, using primers in Table S4B [see www.briggsae.org/polymorph.php and 62].

### RNA interference

Double strand RNA (dsRNA) was prepared as described [17]. Primers to clone the templates are listed in Table S5. RNA interference was performed by injection of young adults, using solutions of 1 mg/ml dsRNA.

### DNA sequencing

PCR products were amplified with primers in Table S4A, purified on QIAquick PCR purification columns (Qiagen), and sequenced. To identify *trr-1* lesions, we used genomic DNA templates.

### Epistasis analyses

At least 20 animals of each genotype were examined, using Nomarski microscopy.

#### 1. *trr-1(vDf3); tra-3(ed24ts)*

*C. briggsae tra-3(ed24ts) XX* mutants are self-fertile hermaphrodites at 15°C and Tra pseudo-males at 25°C [30]. We crossed *tra-3(ed24ts)/+* males with *trr-1(vDf3) cby-15(sy5148)* females, picked individual *XX* L4 cross progeny to new plates, and shifted them to 25°C. From plates that segregated Tra pseudo-males, we identified F_3_ Cby progeny; if no recombination had occurred, these should be *trr-1(vDf3)* mutants, and 25% of them should be *tra-3* pseudo-males.

#### 2. *tra-2 trr-1*

The *tra-2* and *trr-1* genes map very near each other on LG*II*. Thus, to make the double mutant, we picked individual *dpy(nm4)* + *trr-1(v76)* +/+ *tra-2(nm1)* + *cby-15(sy5148)* animals from a balanced strain, and screened their F_1_ self-progeny for non-Dpy females, which might be + *tra-2 trr-1* +/*dpy* + *trr-1* + recombinants. Each F_2_ female was crossed with AF16 males, and *XX* L4 cross-progeny were picked to new plates, and screened to identify those that segregated 25% Tra pseudo-males. These Tra pseudo-males should be *trr-1 tra-2* homozygotes. We also tested *cby-15 trr-1 tra-2(nm1)* animals derived from the non-complementation screen.

#### 3. *trr-1; tra-1(nm2)*

In *C. briggsae*, *tra-1(nm2) XX* animals are fertile males, which produce sperm when young and switch to oogenesis as older adults [30]. Thus, we crossed *trr-1(vDf3) cby-15* females with *tra-1(nm2) XX* males, and picked F_1_ *XX* larvae to new plates. Then we screened the F_2_ Cby self-progeny for males; these individuals should be *trr-1(vDf3) cby-15; tra-1* mutants. We used the same method to construct *trr-1(v76) cby-15; tra-1(nm2)* animals. Finally, we also crossed *trr-1(v76) cby-15; tra-1(nm2)/*+ females with *trr-1(v76) cby-15/+ +; tra-1(nm2)* males, to produce *trr-1(v76) cby-15; tra-1(nm2)* mutants that came from homozygous *trr-1* mothers.

#### 4. *trr-1; fem-2* and *trr-1; fem-3*

Two methods were used to study the interaction between *trr-1* and either *fem-2(nm27)* or *fem-3(nm63)*, which have no phenotype on their own in *XX* animals [22]. In these experiments, we separated individual progeny, so that we could confirm that animals were female or hermaphrodite by assaying self-fertility.

First, *Cbr-trr-1* dsRNA was injected into either *fem-2(nm27)* or *fem-3(nm63)* mothers, and self-progeny were scored using a dissecting microscope. Second, we crossed *trr-1 cby-15*/*+ +* males with *fem* hermaphrodites. Individual F_1_ males were then crossed with *dpy(nm4)* hermaphrodites, and we used the PCR to identify *dpy(nm4) + +/+ trr-1 cby-15; fem/+* animals among the F_2_ progeny. From them we obtained *dpy(nm4) + +/+ trr-1 cby-15; fem-2* animals, and we scored their Cby children to determine the phenotype of *trr-1; fem-2* or of *trr-1; fem-3* homozygotes.

### RT-PCR

For each genotype, groups of five L4 or young adult worms were collected and processed for RT-PCR as described [17]. Two independent samples were used to confirm reproducibility. PCR reactions were run for 32 cycles, using primers listed in Table S4C, and the conditions were shown to be in the linear range for *fog-3* by testing serial dilutions of template.

### Western blot

Samples were made from 300 animals that were hand picked and added to 30 µl 2× SDS-PAGE buffer and boiled for 10 minutes. SDS-PAGE was run using the Bio-Rad mini-protein system II. Half of the sample was loaded to each lane, unless indicated otherwise. Gels were transfered to Immun-Blot PVDF membranes (Bio-Rad #162-0177) and blocked with 5% non-fat milk. Anti-TRA-1 antibody was a gift from Dr. David Zarkower, and was diluted to 1∶1000–2000 in 5% non-fat milk. Secondary antibody was HRP Goat anti-rabbit (Bio-Rad #170-5046), and was diluted to 1: 10000 in 5% milk. Finally, the Immun-Star HRP chemiluminescence kit (Bio-Rad) was used for detection.

## Supporting Information

Figure S1 Sequence of the *Cbr-trr-1* mRNA. The SL1 trans-spliced leader sequence is in parentheses, the 5′ and 3′ UTR regions are gray, alternating exons are red or blue, the cleavage and polyadenylation site is bold and underlined. The 8^th^ exon of wormbase is actually two exons and a 42 bp intron; the 11^th^ exon of wormbase is actually two exons and a 48 bp intron, and the 17^th^ exon in wormbase is actually 144 bp shorter. The correct cDNA has 22 exons, just like *C. elegans trr-1*.
(DOC)

Figure S2 Isolation of *Cbr-trr-1* alleles by non-complementation. We screened for new *trr-1* alleles on the *Cbr-cby-15 Cbr-tra-2* chromosome, following EMS mutagenesis. New *trr-1* mutations are red, and comments are blue.
(EPS)

Figure S3 *C. remanei trr-1(RNAi) XO* animals are Fog. A. Wildtype *C. remanei* PB4641 *XO* male. The spermatheca is boxed in red, and an expanded view shows abundant sperm and no oocytes. Among the eggs laid by 16 mated females, a total of 526/2676 failed to hatch, giving an embryonic lethality rate of 19.6%. B. *C. remanei trr-1(RNAi) XO* PB4641 male. The spermatheca is boxed in red, and an expanded view shows abundant oocytes, demonstrating that the germ line is feminized. From 16 mated females, 870/1824 eggs failed to hatch, for an embryonic lethality rate of 47.4%, confirming that *Cre-trr-1* controls embryonic viability. Moreover, of 65 males scored with DIC optics, 21 made only oocytes, and 32 made sperm and oocytes, for a total Fog rate of 81.5%.
(EPS)

Table S1 Alleles of *trr-1*. The mutagens were Ethyl Methane Sulfonate (EMS) or Tri-Methyl Psoralen with ultraviolet radiation (TMΨ/UV). Fog indicates that homozygotes make oocytes instead of sperm, and Emb indicates that homozygotes die as embryos. Not all molecular lesions were identified. “FS” indicates a frame shift, “n.d.” is not determined, and “Non-Comp.” indicates a non-complementation screen.
(DOC)

Table S2 TRR-1 is required for embryonic development. A. Analysis of homozygous *Cbr-trr-1* mutants from homozygous mothers. B. Analysis of heterozygous *Cbr-trr-1* mutants. All Cby larvae were homozygous for the marker *cby-15*; we suspect that the single Cby larva in the *v108* cross was a recombinant. Error represents a symmetrical 95% confidence interval, calculated for a proportion.
(DOC)

Table S3 TRR-1 acts near the end of the *C. briggsae* sex determination pathway. Epistatic interactions between *Cbr-trr-1* and A. *Cbr-tra-3*, B. *Cbr-tra-2*, C. *Cbr-fem-3*, and D. *Cbr-fem-2*. The construction of each genotype is described in the Methods. The *tra-3* strains were scored at 25°C and the other strains at 20°C. Ψmale indicates an *XX* animal with a male body and defects in the tail that produces only sperm, Fog Ψmale indicates a Ψmale that produces oocytes, either exclusively or after first making sperm. Fog indicates an *XX* hermaphrodite that produces only oocytes. Lethal includes both dead embryos and larvae. In D, line 3 only the Cby or dead progeny from *cby-15 trr-1 +/+ +dpy(nm4); fem-2* mothers were scored. (* 3 of these 14 Fog animals had only small, oocyte-like germ cells rather than large oocytes).
(DOC)

Table S4 Primers used. A. Sequencing primers. B. Primers for analyzing SNPS. The name of the SNP is included in the name of each primer pair. C. Primers for RT-PCR analysis.
(DOC)

Table S5 Primers used for RNAi. The 5′ end of each primer contains T7 promoter sequences, to simplify transcription of RNA from the PCR product. These T7 sequences are capitalized.
(DOC)
