# Supplementary material for: Evolutionary Change within a Bipotential Switch Shaped the Sperm/Oocyte Decision in Hermaphroditic Nematodes
Source: PLoS Genet. 2013 Oct 3;9(10):e1003850. doi: 10.1371/journal.pgen.1003850 (PMC3789826; doi:10.1371/journal.pgen.1003850)
Supplement: Figure S1 — Sequence of the Cbr-trr-1 mRNA. The SL1 trans-spliced leader sequence is in parentheses, the 5′ and 3′ UTR regions are gray, alternating exons are red or blue, the cleavage and polyadenylation site is bold and underlined. The 8th exon of wormbase is actually two exons and a 42 bp intron; the 11th exon of wormbase is actually two exons and a 48 bp intron, and the 17th exon in wormbase is actually 144 bp shorter. The correct cDNA has 22 exons, just like C. elegans trr-1. (DOC) [file pgen.1003850.s001.doc]

**(SL1:GTTTAATTACCCAAGTTTGAG)**aaggaagtgagttcaatctaaatcttcctgtgtatccaaaatccaagtgctggtcactaaaaaaatATGGATCCTTCGATCCCGTCAACGAGTCATCGATCCGTGCCGCCAGATAGGGGCGTACAACCTGATAGAAACCTTCATGTTCAAGAGTTGGAAAACAGGATCCAAAGTCTTGTTCATGGTGGACAGCGAGATGATGTCAAGTTGAAAGAACTTCAAGATATTTGGGCATCACTGGAGAATCACTTCACTGCGAGCTCACATGAGAAAGTCGTTGAAAAGCTGGTTCTCTCCATTCTTCAATTATTCTGTAATACTAGTCCTCAATTCATTTCGGAGAATAACACACAGATGCTTCGCAAATTGATGTTGGAAATACTGCTCCGACTTTCAAACACTGATCCAGTGAAAACGCATAGCAAGGAAATCCTGAAGCAAATGATGAGACTGATTGGAGTGGAGAATGAAGAAAATGCAATACTCGCTATCAGAATTCTCATCGATCAGGGAAGATATGGAAAGCTAGATTACTGCCGAGAAGTTCAATCGCTTTTGCTGTTGATGCGAACAATGGTGAAAGAATTGGCTGAATCTGGTCGCACAGCCGAAATGTTTCTTGTCCGAGAGCTCACAGTACCACCAGCAACCAGTTCTGAAGAGCAATTGATTGCAGAATATCTGACCAAGTGTTACTATGCTCAACCAGTTATTCTGAATGCAAAAGATGGACTGCAAGGTCCCAAATTTAACATGATTCCATCGGCTCATCAATCTATAAAAGTTCTTTTGGAAATGCCGTTTCTCGTCATATTCTTCTATCAGAACTTCAAAACGACAGTTCAAACCGAAGCTTTAGAATTCACACGGCTATGTCTGGATTTTCTGAATGTTCCCGTTCCAGCCGATAAGACCAAATACCATGACGTTCTCACTGATGACTTCGTCACAGTTCAATCTAAAATTCTGTCGTTTGTGAACATTATGGCCAAGATCCCAGCTTTCATGGAGCTCCTGCAACAAAATGGAGACTCGTTGGTGTCCGGAACGATGCAAATGCTGGAAAGATGCCCTCCAGATCTCATATCAGTCCGCAGAGAAGTGTTACTGGCTGTAAAGTACTTTACAGCAGGAGAAATGAAGTCAAGATTTTTCACAATGCTTCCACGGCTCATATCAGAACATTTTATTTTGGGAACCGGGTTTACAGCGATTGAACTTTTGAGAGTTTTCATGTATCAAATGTTGGCCGACCTTATGCATCACACTCGCGATACAATCAGCTACGAATTGATCTCTCATGTGGTCTTTGTCTTCTGTCGGGCACTTCATGACCCGAACAATTCTGCCCAAGTTTTGATCATGTCTGCGCGTCTTCTTAATTCGCTTGCCGAATCTCTCTGTAGAATGGAATCTCAAGCTCCGATCAGAGACCTCATGCTGGAAATCTTGGAAGCACAGGTATCGAAACTGAAGGTTATGGCCGTATATCACATTCCAATTCTTTTCCAACAATATGGAACAGAAATTGAATACGAATACAGAAACTATGAGAGGGAGAGTGAAAAACCGAAAGTCAATGTCATGAAGGAGAGTACCCAAAGAGAAGTTCCTAAACGGAGAACCCGAAAACTTTCGATGGATTCTGTAGAAGAACTCGAGTTTCTTGTAACCGACAATGTGAATATGACTGAAAGTGAGCAGAAGCGCAACGAGCTTCCAACACCAACAAAAGAGCATACAAAGAAAACATCACCAGAGGCGATTCTAAACAGTTTGTATGCAGCATCTACTCAGCCACTAGGATTAAGTGAAACTCGGAACCTTATAAAGTACGTAATGCACACATGCAAGTACGTAACTGGTCAGTTGAAAATCAGTCGGCCTTCTACTGAAATGTACCATTGTGTGAGGGAACGAGATCTTTACGAACGACTACTCCGCTATGGAATCATGTGTATGGATATATACGTTTTGCCAGCAGTTAAGAACCAAGCTCAAGCACATGCTTCCCAGCGAACAAAGGAAGAAAAGGAGGCTTTGGAATCGTTAGCCAATGTTTTTACAAGCATTGATCATGCTATCTTCCGAGAACTTTTTGAGAAATACATGGACTTTTTGATCGAACGAATTTACAATCGAAACTATCCCCTTCAACTGATGGTTAACACTTTTTTGGTTCGCAACGAGGTTCCTTTCTTCGCTTCTACCATGCTATCGTTTTTGATGTCTCGTATGAAGATGTTGGAAGTGAGCAGTGACAAAACCGCGCTGTATGTGAAGCTGTTCAAAATAATCTTCTCAGCAATCGGAGCCAATAATTCGACCATCTATCAAGACAGAATGCTCACAAATTATCTTCCCGAGATTTTGAAACAATCGACCGTATTGGCATTGACTGCTCGTGAACCTACAAATTACTTCTTGCTGCTTCGTTCCTTATTTAGAAGTATTGGAGGTGGTGCTCAGGACATGTTGTATGGAACTTTTCTTCAATTACTGCCAAATCTTCTTCAATTCCTCAACAAGCTTACTTCCTGTCAACATCGGATACAGATGCGCGAGATATTCGTCGAATTGTGCCTGACAGTTCCAGTTAGGCTGAGCTCTCTTCTTCCATATCTTCCACTTCTCATGGATCCTCTAGTTTGTGCGATGAATGGAAGTCCGAACTTAGTGACGCAAGGCCTACGAACGTTGGAATTGTGCGTAGATAATCTTCAACCGGAATATCTTCTCGAGAATATGCTCCCTGTTCGTGGTGCCTTGATGCAAGGCCTCTACCGAGTCATCTCGAAAGCTCCCGACACTGCTTCGATGAAAGTTGCGTTCAGAATTCTCGGAAAGTTTGGAGGAGCCAATAGAAAGCTTCTCAACCAACCACAACTTCTCCAAGTTCGACGCTTTCCCGATTCTTATCTCAATATGGAGTTCTCTCAAATGGGACTTGACGGAAATCACAGTCTACACTTGCCAATTTCCGAGCTGATGAGAGTTGCTGCTGATCAGATGAGATATCCAGCTGATCAGATTTTCAATCCGAATCCTACAAATATACCTTCACCACATGTGAAAAAGTGCTGTATGGAATTGTCGAAAGCTGTTCTACTCGCTGGTCTAGGTTCATCAGGAAGTCATACGGTCCCCACGAAGGATCTTCCAAAAGTGTTGAAGAAATTGCTGACTGGGTTCAATGTTAATCAACGAACAACTGAAATCTACATTTGTCCGAAAGAAAACGATCGAGAAGTTTATGTTAATGCACTTCTTGTCGTGGCTTATGGAATTTGGAATAAAGACGGGTTACGCCAACTCTACAGTAGATTTTTCGTCAAGATCATCCGCCAATTCGCTCTTATGGGCGCAATCGAATTCGTCAGCGGAAATGGTTGGATGCAAAATGCCGACGAGGAGGGTGCTCTTCCACTCTGCCTTGATTCATCAGTACTCGTGGATGCTCTAATCACTTGTCTTTCGGAAACATCAACGAGCTTCTTCTATGGCGGAATCATGTGTCTTCGTTATATCAACGAGACTCTCGAATTGGCTTTACCAGACATCAACCAAATGTCGAAAGTTCCACTGTGCAAATATCTGATGGAGAAAGTGTTGAAACTATGCCATGGACCTGCTCAGTACGCAAGAGCTGGCGGAATTAACGCCTTCATGTATATGATTGAGCACTATCCTCGCAAATTCATCATGGACTTTGTCATCGATGTTGTTGACGCGATTATGGAAGTCCTTCTCGGGCTGGTAGAGGAAATTTCAAGTGGATCTGCCGATATTGCAACTGATTGCTTCAAAAAGATGATGCGAACTTATTTCATTCAAGAAGAAAATCAAGAAGAGGAGAATCTGACATTAGCTTCTATCTTTGTTGCAGTCTTCGGAAAGCACTACTTCCATGGAAACGACAGAATCAGAGATTACATGGCGCAACTTATGGAGTACTGTATGATTCAGTCTCGGCTCGAAACCAATCTAGACAAATTCTACTATCGGTTCAGAGAGTTTTTCGAGCCAGAGTTAATTCGAATCATGGAAACACTGCCAACAATGTCACTTACGGACGCTCAAGGAAGTCTCGATGGTCTTCAACGCTTTGTCTTCATTTGTCCAGAAGGTTTCGAGTTCGAGAAAGATTCGGAAATCTACAAAAGATATTTGGTTCATCTACTCGATCTTGCTCAAACTGATACTCAAACCATCAACCAACGAAATGCTTTCAAAAAGTGTGAGACCTGTCCATCTCATTTCCTTCCTCCGTTCCCGATCCTTCATCACATCGATCAAATGCGTGGAAGTGCTCTCCAATGCCTTGTTATTGCCTACGATCGACTGAGAAAACAGTTGGAAAATACGACGAGAGATATAGAAGATGAACAACTGATGAGTGAGATTCTCGCAATGAATTCTCCCAGGATCACCGTGGAACAAATATTCGAAAACAATGAATCGTGGAGAAGATTGATGACAGTTCTGTTAAGAGCAATAACTGACAAAGATATACCTGATATCGCTGACAAACTTTATCCAGCACTCATGAGAGTAACGCCAGTTCCAACCAACATTATTGCGACTTTTGGAGCCAATTACATTCGTAATATAAGTCGAGCTAATGATGAAAATGACCCGGATCGTACAATCACTTATCATGACTGCCGAAAGTTCAGCATTCTCGTTGAACTGAATCCCAAAATACTGGTGAGAAACATTGTGAAAAATCTGGCCAATCATATTATTAAATACAATATGAGCGACAGTATCTCGAATATTTTGGTAATGCCAAATGAAGCAAAAGAGGAAGAAGTGGAAGCATACGAAGCTGAGAAGAAGAGAGGAGTTCGAGATCTGGAAATGATTGGATACACTGCAAAAATGCTTGCTGGTTGTTCGATGGAAATTCTAACTGCTGAAATAATCATTGATATTACTCGTTTTGCCGCTAAATTCGAGTATACCTATTCACAAGATGTTCTACCAAATTGGATCGATGATGTAGTGAAGTTGATGAACAAGGCTCCTGTTGAAGTTTGGAAGTTTTTCTTGTTACGCGAATCAGTTGCCAATCCTGCTCGACGTTCTCTTATCCGAAGAGCCATAATCTTCCCTACTAGTGAGCCTCTGCGAAAAGTTTTCATGCAAACTCCAGAGTACTTGGAACGACTGATAGATTCAAATCTCGATAACTATGACAACTCTGATGAACGTGTCATCATTGATCGCGAAATGTTCCTGCTCTCGTTGGTGGACCGAATCTCCAGAAACTGTCACGACTGGCTCTCAGACCCAAGTTTGTCCCCTATTCCTCAGTTGAGAGCATTTTTCAATGGAACTGAGTTCATGGATAGATACTCCGTCAGGTCAATAATGGTGGAAGAAGCAAGAGAGATTCGAGTGATAAGTATGACAGAAGATAAGTACAAAGTTCCGAAGTTGATGACCAATATCTTTTTGAGATATCTCAGAAATAATATCCAAGACTATGATATGTTCTTCAATGTAGTATCGGTCTTCATGGGAAAATTCCAAACCGATTTTACATTCGTCCGAGAATATCTGGAAGTTGAAGTCATTCCAAAAATGCCACTATGGTGGAGAAGAGAGATTTTCATAAAAGTAATGGTGATGTTCGAAGAAAATGCGCAGAAGGCATGCAAGGATTTCCGGATTCTAAAAGCTCTTCAGTACCTGATTCTTCCATCTCTACAATGGGCGTTCGAACGATACGATACCGATGAAATAGTTGGAAGTGCGCCTATAGACGATTCTGAGAATGCAGCAGATGCGGAATCTTCCAACAACACCGAAAATCTTGTCGGGCGTCTCACATCAGTGATCGGAGCGCACCGCCTGGATTTCAGCGATGGAATGATAATTTTGTTCTATCAATTGTGCACTCTATTCGTTCAGCATGCACCTGAGCATATTCACAACAATCATTGTAAGAAACAAGGTGGACGTCTTCGGAACTTCATGCTTTTCGCTTGGCCGTGCCTTGCCACTCCGAACCGTCAGGACCCGACTTTGAGATACACTGGATTCTTCTTCCTCGCGAACATAATCGAACGATTCACTATAAACCGGAAAATCGTTCTACAAGTGTTCCAACAACTCATGACAAACTATCAGCAAGATACGAGAGATCAAGTTCGTCGAGCTATTGATATTCTGACTCCGGCGTTGAAAGTCAGAATGGAAGACGGACATCAGCAGATTCTAACCCAAGTTAAAAAACTGCTTATCGAAGAAGGCCATATTTTGCAGCATATTCAACATATTCTAGGTACAATCATTCGTAACTGGCGGGTTTACTATCATATTCGTCATGAGATCCTCACTCCTCTTCTGAATGCAGTTCAACGAGCCCTCACCATGCCAAACAGTGTTATTGAACACGCTCAAACTCGAAAACAAGCGATCGAAGTCTGTGAAATGATCATCAAGTGGGAGCTGCTCAAACTACACAAGACAGATCATATCATCACTGACGATGAGGCTAATGAAGTCGATAAACTATACGAGAAACTTCGTGGTGCTAGTAGTCCGGATAGGTACGATTTTGAGGATCAGCAAATGAAAAAGGATCTTCTCGACTCTCAACGTGTTATCACGAGAGAACATGTGGACATCGTTGTGAATATGCTGATGCGGTTCTGTGTGATGTTCCATACAAGTGCCCAGAACAATTCCACTTCTGGACAACAAGGTGCCGAGCTAGTCAAGAAATGCCAGTTGCTCCTCCGAATCTGTCTTCGTTCGAGTGTTTGGGGAGACTTTGTCAACATTCGAACTTCGATTCTCAACAACTACATCGTTGTTCCGTCGGAATTGATCCCGAAACAAAATGAAGTCCAAAACCCGGAGTACGTGCTTGCTGCTAACAATTCTCAATATACCATCGAAATGCTGAATGTTATCGTTCCAATTCTGCCGAAACCCACTTTGAAGAACGTTCTTAATATATTGCAACCAGCACTTATTGGTGTTATTCAGTCTAGTGGTCATATGTCCCGAGGAATCACACAGTTGATTTCTCGGCTAGGTGAAAGAACCAGTGTTTCAACAAACGGCTTAGATGAATTCGAATTGCTCAATTCCTACATAGTTAAATATATTCATGATAGTTTCTCAACCATTTTGAGAAATCAAAACGCGCCAGTCTTGAGTGTTCTCGGTTCATTCACTCTACTTCGAGCAATGTGTGGCCACGAGGCAGGATTCTTGGACAATTTCATGCCAACTTTCCTCAAAGTTATGGATAGAGTGGCGAGAGAGCACCTGCAGTTCAATAGCAGACAACAACCATCAGTACAGAAAAATCTATCTGAGTTGACGTGTGTATGCATGGAACTGGTGCGCCAAAGAATCGATCATATCGGCTTGGAGTTGAAACGAACAACGATAACCGATGTCATGACAGAGCTTATCTTCAAGTCAACAAGCGAAAGGGTAATCCAAGTATGTGCGAAGCTGATAGGAGCCATGCTGTCTCCAACCGACATGGAATTCTCACTTCATACCTGTCTTCAACAACTGGTTCGAATTCAATCAGTGATCATAAGCAAGTTCAAAAACTGTAAAGAAGTCATTACGGAGTTTCTTGTCGTTGTTATCAAGGTTTTTGAGAACGCTGAATATCGAAACTCGGAATACGGAGCTCGTCTGTGGGAGGCATTCTTTTGGGGTCTTAAGAGTACGGATCCAACGACAAGAGATAGTTTCTCAGCCGTCTGGGAGATGACGTGGCCACAAATGTCAACTGCTGATATATGTCACAGAATGAAGTACATCATGAAGCACCAAGATTGGTCGAAGTTCAAACATGCGTTTTGGTTGAAATTTGCGCTATGGGGAATGTTAAGAGCAATTTCTAAACGACCAAAATCTGTGAACAATCCAAAGAAGAAAGTGGTGATGTTGAATTGTGCAACTCCGTGGAGGACTATTGAATATGCTGCAAGACTCAAAGAGCAACATATGGAAACTGATCCGATGATTAAGCTTGAAGAACCAGAACCAATGGAAGTCGATCAGCCGAAAAACGCTCCAGCGGAAGAGCCGAAAGACAATAAATTGTCATTGGATGATTTTCTAGCTGGTCAACAGGAACTTCTCGAGGAAGCCGCGGAATTCGACTTTGCTGATGCTCTAGATACAGTCTCTCAAATCACATTTGGAATCAATGATAACGGAATGACTAGTAGGATCTGGGTGACCTTTTTCAAATCGTTCTGGGCATCACTACAACCGCGAGAAGTCGAGGATTTCACTGCGTTGATTGTGCCGTTTTTGAGCAGTGGAGTTCATAATCAATTCCAAACTGGTGTACAAGACAGTGTTCTGGCTGTTTGGCTTGAGGCGATTGGTGAAAAAGTACCATTGCCATCAAGTTTGATCGAGTTCATCTCATCAAAGCACGAATGTTGGTATACGGGAATTTCAATCCTCGAAAGCAGTATTTGGTCCATACCAAAACAACTCAATAATACTCTCCTAGGCAACATCAATTGTGATCGTTCTCTGACTTCCAATATCGAGACTCTCGAATCATTAGGAGCTTTGTACAAGGAATTGGCGGAATTCGACCAGTACTCTGCAATCTGGGAACGTCGTTCCGTCTTCCCTGAAACAATGAAGGCGATGTCAGCATTACAATTGGGTGATATGGACACTGCTGCAAGCATTCTCGAACAAGCGATGAATAAAGAAATGGAACATCTTCCTGTACCTACGGCTAATGCTGCTCCACCCGGCCCTAACGACAGACAAATCTCGCCAATCTATGACCGGGAGTATGAACAATGGATGCAAATGTACATGTCGAGCTGTTCCGAATTGCTTCAGTGGCAAACTGTCGCCGAAATCTCCAACAGCAGAGAAGTGCAAGATGTTCGAGGAATAATCACAGCTGCTTCGCATATTCCGGATTGGAATCTTGTCGAAGATTGCAGAAGCATGTTGTCTGGTTGTATCCCTCCAGACTTCCATTTAGAATACACTGTTTTCAATTTGATGAGTACTGTTATGAGATTGAATGAATCTGTGAATGTGCCTCATGCTCGGGAAAGATGCAAACAAGCGCTTCAAGAATGTATCGAAGCCCATATCAGCCGATTCAGAGCACTTCCGTCTGTCACCTCTTATGGCCACGTCAAGATTCTTCAATCTATGAATTTGGTCCGAGATATAGAAGAGTCGATGGAAGTTCGGATAGCTCTTCTCGAACAGCCAACCAAAATGGATCAAAGTTTGATGATGGACATGAAAAGTTTAATGAAAGTTTATCGGAATCGCACACCAACCACGGCTGATGACATGGGATTCGTTGCGACATGGTACGATTGGAGGAATCAGATTCATGGGATGATGCTTCAGAGATTTGAGTGGTTCGATAAATCTTCGCTCAGTACTACCGGTAACGGTAATCAATCAATTGTTCCGATCCATTCCATGGCTCAAGCACAGCTGACGGTGGCTAAACACGCAAAGAGCTTAGGATTCAACAATCTCGCCAAAGATCTTTTGAACAAACTTGGAGGTTTGCCAGCTATCCCAATGATGGATGCAGTTGATAAAGTTTGCACATATGGAAAAACACTTCGTGCATTGTCTAATAACGTCGATGATGAGAGATCGAAGCAGGAACTTTTGTATGAGGCTCTTGAAGTGCTTGAAGACGTTAGAATCGATGATTTGCAAAAAGATCAAATTACCTCACTCCTATTCAATAGAGCTACAATTCATTCGGCTCTCGGACAAACTGCGAACGCTGATCGAGCTTTCTCGGCGGCTGTACAATTGACCGACATGAAAACTGCAAACGTTCCAACGGGAATAAAATTGTTCCGGCAGTGGGGTAATCATCTGAATAAGCTCTTCTTCGATCAGTCCCAGATGGTATCCAAAGAAACGAGCGAAAACTTCGGTAGACAAGCTCTTTCCTGTTATTTCGTCGCTGCACGTGTGGATGGTGACCTCAAAGCTCGGAAACCTATTGCTAAAATTCTGTGGATAGCGAAACATTTGATGGCTAGCGGAGCTTCTGAAGCACTGAACAGAGTCATCCAAAAACATCTTCCATCCTTGAATCTCTTCAATTGGCTCTACTGGATACCACAGCTAGTCACTGAAATCAGTCACCAACCAAACAACAACTTTATAATGGTTCTGTGCAGAATAGCTGCTGCTCATCCCCTCCAAGTGTTCTACCATATTCGCGAAGCAGTTTCTGTAGAAGATATTGATGCAGTTTTCGCTCAAGATTATACAGAAGAAGAAATGTCAATGGATACACCTGATGACGAAGCTTTCTCCAACGATCCGCCATTTTCCCGAGCTCTGAAAATATGTCTCAAGTATCGTCCTACAGACATACGCGTCTTGCATCGGATTCTCAAAGAACTCGATCAAATGACAGAGACATGGGTTGAAAGACATTTACGCTTCGCCGTAGCCATCAAAGATCAGTTATTCGAAGATTTTGCTGAACAAATGGATGCTCGTTTCAATGAGATGCAATTCTCAGGTGCTGTATACGAACTAACTCAGAAGTGGAAACGTCAACTTGAAGAAGACTATAAGTTCTTTGAGAATAACTACAACCTGGATCTTTTGGAAATCAGGAACAGACGGAGGGTTATTGTGACGAAGGGATACATGGGAACTGTGCCAAGTCAAATCATGTTCGAAAAAGAACTGAGTCAAGTATTCACTGATCCACCAGAGATGAAGGACGAGTTTGAATATGTTACTGAAATCACGAAAGTAATTTTTGATCAACTAGACATTCGATCACCACAAGCCCCTCGTCCAGCACTTTTCGTTCGTACGGTTATGGAATGGATTCGAATTATTCGTCGTCGGTTTGATAGACTGCCAAGACGTGTTCCTATGGAGATTTCGAGCCCTTATCTCGCGCGATTCAGTCATCGTACTGGTTGCATCGAAATGCCATATGATCTTCTTAATGTGCTACGTGCGAAGAATCACAGTCTAAATGCCACTAATCAAACTGGACAGTACATATCGATGATGTCTCGATTTGAACCGTATTTTGAAATTGTGATGAGAGGCGGACAAGTTACTCGAAAGATCTATCTTCGAGGACAAACTGGAAAAAGTGCAGCGTTCTACCTCAAAAAGTCTATCAAGGATGAGCGAACGAATCGGGTGCCCCAGATGTTCAAGCATGTCGATTACCTTCTTCAGAACGATAGAGAAACTGCGAGAAGACATTTATCTGTCCCATCACTTCTTCAAATGAGAGTCAGCAAGAACACGACATTCTGCGAGATTGCCTCTGTCCAACCGTATGCTATACCGCAAGATTGTTCAAGAAATTATCCAGCATCGCAAATTGAAGTCATGCATCCTTATGAAGTTTTGACATCCACATTCAACGGACTGTACTCTCCAGATGACATGGTTATGCATTTCTATGAAAGATTCGCTGACAGTTGCTCTTCCATCGGACAGCCTCTCCCGCAGAACATAGATCCTTCAATGGCATCGCAACCACGACTTACTGAACCGCACCATGTGAAGAACATCATTTATGAAGACTTCGCCAGGGATATGATTCCATTCCGACTGCTGACTGATTACCTTCTTGCACGCTATCCGGATCCAGTAATGTTTTACGCTATGCGAAAGCAGTTCATTCATAGTTTCGCCGTCCTCTCCATCATCGAATATCATTGCAATCTGTCTCCAATGACTCCTCACCAAATGATAATCTCTATGAATACTGGAGTTCTAAATAATCCATTCTACCGCTTCGAACTTGGAACTGGGCAACTTATGGATATCGAACATTTTGCTCATGAAGTTCCCTTCCGCTTAACGCCAAATTTGATGATGTTCGTTGGAGTTGCTCAGGATGGAGATTTGTTATGGAGTATGGCAGCAGTGGCTAGATGTTTAATGAAAAAAGAACCAGGAGCTGTGATGAGACCATTGCTCTGGGATGAGTACGCCAATAATGTTAATTATGAAAACATGATTTACATTTGCCACGCTGCGAATTCCTATGTCAAGTGCATCGAAAACAAGGTTGCAATGACGAATCGCCATGATGCCAAAGTGAAGAAAGACGACTGCAATTCGCTGATTATCCGGGCCAAAGATTCCGACAATCTGTCTCGAATGCCCCCAACATATCACGCTTGGTTCTAAatctctgtttcatagcttatttccccagaatctctccccagaatccgtgattctaccgtttttgatctcttgttgcatgttttaaccttatattattcaccacatctctttcctttcaatatattggcatttttctttaactaggagtaactatttactgaacgctgtgatctttcattttaacaggtatgtaaataatctagttactctgttatg**aataaa**aattttcaaaaaacAAAAAAAAAAAAAAAA
