# Supplementary material for: Evolutionary Change within a Bipotential Switch Shaped the Sperm/Oocyte Decision in Hermaphroditic Nematodes
Source: PLoS Genet. 2013 Oct 3;9(10):e1003850. doi: 10.1371/journal.pgen.1003850 (PMC3789826; doi:10.1371/journal.pgen.1003850)
Supplement: Table S1 — Alleles of trr-1. The mutagens were Ethyl Methane Sulfonate (EMS) or Tri-Methyl Psoralen with ultraviolet radiation (TMΨ/UV). Fog indicates that homozygotes make oocytes instead of sperm, and Emb indicates that homozygotes die as embryos. Not all molecular lesions were identified. “FS” indicates a frame shift, “n.d.” is not determined, and “Non-Comp.” indicates a non-complementation screen. (DOC) [file pgen.1003850.s004.doc]

| **Allele** | **Screen** | **Mutagen** | **Molecular lesion** | **Coding change** | **Phenotype** |
| --- | --- | --- | --- | --- | --- |
| *v76* | F_2_ females | EMS | C3367T | L1123F | Fog |
| *v104* | F_2_ females | EMS | G3587A | G1196E | some Fog |
| *v106* | F_1_ Non-Comp. | TMΨ/UV | Deletes 3459-3544 | FS after Y1153 | Fog |
| *v107* | F_1_ Non-Comp. | TMΨ/UV | n.d. |  | Fog |
| *v108* | F_1_ Non-Comp. | TMΨ/UV | Deletes 1383-1716 | FS after I461 | Fog |
| *v109* | F_1_ Non-Comp. | TMΨ/UV | n.d. |  | Fog |
| *v111* | F_1_ Non-Comp. | TMΨ/UV | Deletes 1902-2264 | Deletes V636-Y736 | Fog |
| *vDf3* | F_1_ Non-Comp. | TMΨ/UV | Large deletion |  | Fog |
| *vDf4* | F_1_ Non-Comp. | TMΨ/UV | Large deletion |  | Emb |
| *v124* | F_1_ Non-Comp. | EMS | G3587A | G1196E | Fog |
| *v125* | F_1_ Non-Comp. | EMS | n.d. (not *v76*) |  | Fog |
| *v126* | F_1_ Non-Comp. | EMS | n.d. (not *v76*) |  | Fog |
| *v127* | F_1_ Non-Comp. | EMS | n.d. (not *v76*) |  | Fog |
| *v128* | F_1_ Non-Comp. | EMS | G11734A | D3912N | some Fog |
| *v130* | F_1_ Non-Comp. | EMS | n.d. (not *v76*) |  | Fog |
