# Supplementary material for: Evolutionary Change within a Bipotential Switch Shaped the Sperm/Oocyte Decision in Hermaphroditic Nematodes
Source: PLoS Genet. 2013 Oct 3;9(10):e1003850. doi: 10.1371/journal.pgen.1003850 (PMC3789826; doi:10.1371/journal.pgen.1003850)
Supplement: Table S2 — TRR-1 is required for embryonic development. A. Analysis of homozygous Cbr-trr-1 mutants from homozygous mothers. B. Analysis of heterozygous Cbr-trr-1 mutants. All Cby larvae were homozygous for the marker cby-15; we suspect that the single Cby larva in the v108 cross was a recombinant. Error represents a symmetrical 95% confidence interval, calculated for a proportion. (DOC) [file pgen.1003850.s005.doc]

| **A** | *cby-15 trr-1♀*  X  *cby-15 trr-1/++ ♂* | | | | | | |
| --- | --- | --- | --- | --- | --- | --- | --- |
| **Allele** | **WT larvae** | **Cby larvae** | | **Dead larvae** | **Unhatched eggs** | | **Lethality** |
| *v76* | 256 | 164 | | 0 | 82 | | 16.3% |
| *v108* Δ | 186 | 1 | | 162 | 222 | | 67.3% |
|  |  | | | | | | |
| **B** | *cby-15 trr-1*♀ X wild type ♂ | | | | | | |
| **Allele** | **Surviving larvae** | | **Unhatched eggs** | | | **Embryonic lethality** | |
| *v104* | 280 | | 17 | | | 5.7% ± 3.3% | |
| *v76* | 762 | | 120 | | | 13.6% ± 2.4% | |
| *v111* Δ | 292 | | 42 | | | 12.6% ± 4.1% | |
| *v106* Δ | 207 | | 74 | | | 26.3% ± 5.7% | |
| *v108* Δ | 473 | | 254 | | | 34.9% ± 3.7% | |
