# Supplementary material for: Evolutionary Change within a Bipotential Switch Shaped the Sperm/Oocyte Decision in Hermaphroditic Nematodes
Source: PLoS Genet. 2013 Oct 3;9(10):e1003850. doi: 10.1371/journal.pgen.1003850 (PMC3789826; doi:10.1371/journal.pgen.1003850)
Supplement: Table S3 — TRR-1 acts near the end of the C. briggsae sex determination pathway. Epistatic interactions between Cbr-trr-1 and A. Cbr-tra-3, B. Cbr-tra-2, C. Cbr-fem-3, and D. Cbr-fem-2. The construction of each genotype is described in the Methods. The tra-3 strains were scored at 25°C and the other strains at 20°C. Ψmale indicates an XX animal with a male body and defects in the tail that produces only sperm, Fog Ψmale indicates a Ψmale that produces oocytes, either exclusively or after first making sperm. Fog indicates an XX hermaphrodite that produces only oocytes. Lethal includes both dead embryos and larvae. In D, line 3 only the Cby or dead progeny from cby-15 trr-1 +/+ +dpy(nm4); fem-2 mothers were scored. (* 3 of these 14 Fog animals had only small, oocyte-like germ cells rather than large oocytes). (DOC) [file pgen.1003850.s006.doc]

| **A.** | **Ψmale** | **Fog Ψmale** |  |  |
| --- | --- | --- | --- | --- |
| *tra-3(ed24ts)* | 100% | 0% |  | (Kelleher et al., 2008) |
| *cby-15 trr-1(vDf3); tra-3* | 0% | 100% |  | n=27 |
|  |  |  |  |  |
| **B.** | **Ψmale** | **Fog Ψmale** |  |  |
| *tra-2(nm1)* | 100% | 0% |  | (Kelleher et al., 2008) |
| *tra-2(nm1) trr-1(v76)* | 0% | 100% |  | n=21 |
| *tra-2(nm1) trr-1(v125)* | 0% | 100%* |  | n=14 |
| *tra-2(nm1) trr-1(vDf3)* | 0% | 100% |  | n=20 |
|  |  |  |  |  |
| **C.** | **Hermaphrodite** | **Fog** |  |  |
| *fem-3(nm63)* | 100% | 0% |  | (Hill et al., 2006) |
| *trr-1(RNAi); fem-3* | 29% | 71% |  | n=164 |
| *cby-15 trr-1(v104); fem-3* | 0% | 100% |  | n=101 Cbys |
| *cby-15 trr-1(v128); fem-3* | 0% | 100% |  | n=56 Cbys |
| *cby-15 trr-1(vDf3); fem-3* | 0% | 100% |  | n=20 Cbys |
|  |  |  |  |  |
| **D.** | **Hermaphrodite** | **Fog** | **Lethal** |  |
| *fem-2(nm27)* | 100% | 0% |  | (Hill et al., 2006) |
| *trr-1(RNAi); fem-2* | 0% | 39% | 61% | n=464 |
| *cby-15 trr-1(vDf3); fem-2* | 0% | 58% | 42% | n=230 Cby or dead |
