# Supplementary material for: Evolutionary Change within a Bipotential Switch Shaped the Sperm/Oocyte Decision in Hermaphroditic Nematodes
Source: PLoS Genet. 2013 Oct 3;9(10):e1003850. doi: 10.1371/journal.pgen.1003850 (PMC3789826; doi:10.1371/journal.pgen.1003850)
Supplement: Table S4 — Primers used. A. Sequencing primers. B. Primers for analyzing SNPS. The name of the SNP is included in the name of each primer pair. C. Primers for RT-PCR analysis. (DOC) [file pgen.1003850.s007.doc]

| **A. *trr-1* sequencing primers** | | |
| --- | --- | --- |
| **Primer name** | **Sequence** |  |
| 0209F | cgcaaacgcgccaacctacattat |  |
| 1225R | tgggaccttgcagtccatcttt |  |
| 1078F | ggtcgcacagccgaaatgtttctt |  |
| 2087R | acctgtgcttccaagatttccagc |  |
| 1944F | tgcgcgtcttcttaattcgcttgc |  |
| 2952R | ggtcgaattattggctccgattgc |  |
| 2807F | gcaacgaggttcctttcttcgctt |  |
| 3757R | tgatcagcagcaactctcatcagc |  |
| 3568F | cacaacttctccaagttcgacgct |  |
| 4574R | tgaaatttcctctaccagcccgag |  |
| 4418F | aactatgccatggacctgctcagt |  |
| 5374R | tggttggaactggcgttactctca |  |
| 5223F | tcccaggatcaccgtggaacaaat |  |
| 6184R | ccaccattattgacctgacggagt |  |
| 6036F | aatgttcctgctctcgttggtgga |  |
| 7011R | agctcgacgaacttgatctctcgt |  |
| 6886F | actggattcttcttcctcgcgaac |  |
| 7908R | cgttcttcaaagtgggtttcggca |  |
| 7658F | acaaggtgccgagctagtcaagaa |  |
|  |  |  |
| **B. Primers for analysis of SNPs** | | |
| **Primer name** | **Sequence** | **SNP to be assayed** |
| cb43472F | agagcagtctaatgataccaaaa | *Rsa*I |
| cb43472R | tttttagaatgccaactgtattt |  |
| cb43431F | atactgatggttcagaagatgtc | *Msp*I |
| cb43431R | actaacatctgaagtcgaatcaa |  |
| cb43421F | ttcattctcatgcccgtcgaagac | *Tsp509*I |
| cb43421R | agtcacacccgtttagctgcttct |  |
| cb43389F | atgtgaagagatggatagaaaga | *Sac*I |
| cb43389R | gcttggatcttaatgttattcaa |  |
| cd43333F | aacgttttggtagatgttcaat | *Hae*III |
| cd43333R | cagatgaaagttcaggtcagtag |  |
| cb43262F | aaaaagcacggttaaatcataa | *Dra*I |
| cb43262R | cgttcaaagagatggaaaaa |  |
|  |  |  |
| **C. Primers for RT-PCR** | | |
| **Primer name** | **Sequence** | **Gene to be assayed** |
| fog-3 F11 | agttccgcgaagagcaaccgga | *fog-3* |
| fog-3 R11 | aagatgcgcagctgagttggca |  |
| cbr-tra-1A F1 | cccaagtactgaggatcccgaa | *tra-1* |
| cbr-tra-1A R1 | gctccatgtgttttgactcggaa |  |
| cb-tbb-2 639CF | aacgttgaagctcaccaacccagt | *tbb-2* |
| cb-tbb-2 1056CR | ggcggtcttgacgttgtttggagc |  |
