# Supplementary material for: Evolutionary Change within a Bipotential Switch Shaped the Sperm/Oocyte Decision in Hermaphroditic Nematodes
Source: PLoS Genet. 2013 Oct 3;9(10):e1003850. doi: 10.1371/journal.pgen.1003850 (PMC3789826; doi:10.1371/journal.pgen.1003850)
Supplement: Table S5 — Primers used for RNAi. The 5′ end of each primer contains T7 promoter sequences, to simplify transcription of RNA from the PCR product. These T7 sequences are capitalized. (DOC) [file pgen.1003850.s008.doc]

| Target | Sequence |
| --- | --- |
| *Cbr-trr-1 II* | F: TAATACGACTCACTATAGGGAGAgtttggcttgaggcgattggtgaa  R: TAATACGACTCACTATAGGGAGAttgacgtggccataagaggtgaca |
| *Cbr-mys-1 V* | F: TAATACGACTCACTATAGGGAGAtgtcgaaggatgccggttagtgt  R: TAATACGACTCACTATAGGGAGAgctgaattcgataagcagactgcca |
| *Cbr-epc-1 III* | F: TAATACGACTCACTATAGGGAGAtatgactgcacggcgagagaaaca  R: TAATACGACTCACTATAGGGAGAtttcaccggttccgaatgagacga |
| *Cbr-ing-3 II* | F: TAATACGACTCACTATAGGGAGAatcgcgttgtatcttctcgtgcct  R: TAATACGACTCACTATAGGGAGAgctcacgatggtcatttggtgcaa |
| *Cbr-ssl-1 III* | F: TAATACGACTCACTATAGGGAGAagagagcagcgaaacgagtgagtt  R: TAATACGACTCACTATAGGGAGAacttgtcgatggctcatcgtcctt |
| *Cbr-ekl-4 I* | F: TAATACGACTCACTATAGGGAGAgctgttcaacttgatcgctggcaa  R: TAATACGACTCACTATAGGGAGAttcatgatggactccgaagcgact |
| *Cbr-gfl-1 IV* | F: TAATACGACTCACTATAGGGAGAtggccgaagttattgagcgcatga  R: TAATACGACTCACTATAGGGAGAattcttgctcgttgcactcgttgg |
| *Cbr-ruvb-1 V* | F: TAATACGACTCACTATAGGGAGAatgaagagtatggctggcagagca  R: TAATACGACTCACTATAGGGAGAacaactggagccatcggagattca |
| *Cbr-ruvb-2 IV* | F: TAATACGACTCACTATAGGGAGAcaagctgctgggctcattgtcaaa  R: TAATACGACTCACTATAGGGAGAtcagatggaataccgtgcgctgat |
| *Cbr-mrg-1 III* | F: TAATACGACTCACTATAGGGAGAattcaatcggtccacgagaagcca  R: TAATACGACTCACTATAGGGAGAcgattcgtacatttcggcgccttt |
| *CBG03742 I* | F: TAATACGACTCACTATAGGGAGAagagaacggtcaggaatctgccaa  R: TAATACGACTCACTATAGGGAGAtcagatacagtgacggtcgttcca |
| *Cbr-pcaf-1I* | F: TAATACGACTCACTATAGGGAGAtcttcgtctcaacgccgaaaggat  R: TAATACGACTCACTATAGGGAGAactctgcaccgttctccatcttgt |
| *Cbr-ada-2 II* | F: TAATACGACTCACTATAGGGAGAatgagcatcaatcaaacactgg  R: TAATACGACTCACTATAGGGAGAtcaatcgagggaaatcttgtct |
| *Cbr-taf-6.1 II* | F: TAATACGACTCACTATAGGGAGAcaacgtcatcgtgtctttcaat  R: TAATACGACTCACTATAGGGAGAaatctgcgattcacattcacac |
| *Cbr-taf-9 III* | F: TAATACGACTCACTATAGGGAGActatcaacaggtggaccagaca  R: TAATACGACTCACTATAGGGAGAgagctcttcgcttgagcatatt |
